# Vasoconstricción cerebral fatal, presentación inusual de una enfermedad inusual

**DOI:** 10.7705/biomedica.5774

**Published:** 2021-06-15

**Authors:** Hernán Bayona, María Camila Valencia, Angélica Peña, Natalia Ramírez, Carlos Martínez

**Affiliations:** 1 Facultad de Medicina, Universidad de los Andes, Bogotá, D.C., Colombia Universidad de los Andes Facultad de Medicina Universidad de los Andes BogotáD.C. Colombia; 2 Centro de ACV, Hospital Universitario Fundación Santa Fe de Bogotá, Bogotá, D.C., Colombia Centro de ACV Hospital Universitario Fundación Santa Fe de Bogotá BogotáD.C. Colombia

**Keywords:** accidente cerebrovascular, hemorragia cerebral, vasoconstricción, mortalidad, pronóstico, stroke, brain hemorrhage, vasoconstriction, mortality, prognosis

## Abstract

El síndrome de vasoconstricción cerebral reversible se produce por la constricción variable, segmentaria y multifocal, de las arterias cerebrales y, generalmente, es de curso benigno. Se describe el caso de una mujer de 49 años que consultó por cefalea, síntomas visuales y convulsiones; tres días después, presentaba áreas de vasoconstricción en, por lo menos, dos territorios vasculares y dos segmentos de las mismas arterias.

Fue internada en la unidad de cuidados intensivos para controlarle la presión arterial y recibir tratamiento médico. Tuvo una evolución tórpida y, en el séptimo día de hospitalización, desarrolló edema cerebral maligno, tras lo cual ocurrió la muerte cerebral. Se inició entonces el plan de donación de órganos y, posteriormente, se practicó una autopsia guiada del cerebro. El estudio de patología descartó vasculitis y reveló áreas de hemorragia en la convexidad cerebral.

Se discuten los aspectos más relevantes de los casos con evolución fulminante informados en la literatura científica. El síndrome de vasoconstricción cerebral reversible se asocia con resultados fatales cuando los pacientes tienen una deficiencia neurológica focal, la neuroimagen inicial muestra alteraciones y hay un deterioro clínico rápido. Es importante conocer los factores asociados con un mal pronóstico, y establecer estrategias tempranas de intervención y prevención.

## Abstract (en)

The reversible cerebral vasoconstriction syndrome is a variable, segmental, and multifocal constriction of brain arteries, usually with a benign course.

We describe the case of a 49-year-old woman who presented with headaches, visual symptoms, and seizures. Three days after admission, vasoconstriction areas were found in at least two vascular territories in two segments of the same arteries.

The patient was admitted to the intensive care unit where her blood pressure was monitored and she received medical treatment. Surprisingly, the patient presented an unpredicted evolution in developing malignant cerebral edema on the seventh day after admission. She then suffered brain death and was taken to organ donation. A guided nervous system necropsy was later performed. The pathology discarded vasculitis and exhibited hemorrhage areas in the cerebral convexity.

Herein, we discuss the most relevant aspects of cases with fulminant evolution reported in the literature. The reversible cerebral vasoconstriction syndrome is usually associated with fatal outcomes when patients exhibit focalization, their first neuroimaging typically shows disturbances, and a rapid clinical deterioration occurs. It is crucial to identify factors linked to poor prognosis and set intervention strategies and early prevention.

El síndrome de vasoconstricción cerebral reversible se produce por una vasoconstricción variable, segmentaria y multifocal, de las arterias cerebrales (1).

Esta condición patológica es más común en mujeres entre los 10 y los 76 años, con un pico a los 42 años (2). Hasta en el 70% de los pacientes puede haber factores precipitantes (3), entre los cuales se han mencionado estrés emocional y físico, actividad sexual, puerperio, trauma, maniobra de Valsalva, y uso de sustancias vasoactivas o de inhibidores selectivos de la recaptación de serotonina (4-6).

Los hallazgos clínicos son diversos, pero se sabe que la forma más común de presentación clínica es la cefalea “en trueno” (7). La principal herramienta diagnóstica es la angiografía cerebral, considerada la prueba de referencia, aunque no es el único estudio de imagenología que se puede utilizar como método de evaluación (3,8).

A pesar de que muchos casos se resuelven de forma espontánea, algunos pacientes pueden desarrollar complicaciones como hemorragia, convulsiones e infartos cerebrales (3), e incluso, se han reportado casos fatales asociados con este síndrome (9-12).

Se presenta el caso de una paciente que falleció. Se describe la secuencia de eventos clínicos que llevaron a su muerte, haciendo énfasis en aquellos factores que deben alertar sobre un posible curso fulminante.

## Presentación del caso

Se trata de una paciente de 49 años, diestra, que consultó al servicio de urgencias por un cuadro clínico de dos horas de evolución de cefalea pulsátil de inicio súbito en la región occipital, con intensidad de 10/10 en la escala análoga visual, acompañada de náuseas sin emesis, fotofobia, fosfenos, visión borrosa, mala diferenciación de las figuras, diaforesis y parestesias de las manos.

Como antecedente, la paciente refirió haber sufrido de migraña desde hacía más de 30 años, la cual se trataba con propanolol de 40 mg tomado diariamente de forma profiláctica; ocasionalmente, tomaba naproxeno. Tenía alergia a los medios de contraste yodados y un hermano había sufrido un aneurisma cerebral.

A su ingreso a urgencias, su presión arterial era de 154/88 mm Hg, con modulación del dolor en 5/10 en la escala análoga visual. Una hora y media después de su admisión, presentó una crisis convulsiva con desviación de la mirada hacia la izquierda, movimientos tónico-clónicos en los miembros superiores y mordedura de la lengua.

**Figura 1:**
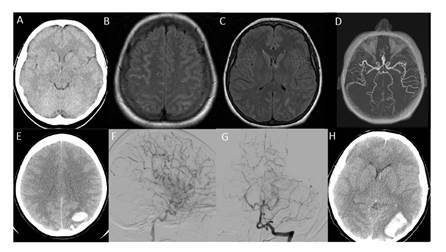
Estudios de neuroimágenes. **A.** Tomografía computarizada cerebral simple, reportada como normal. **B-C.** Resonancia magnética cerebral en la que se evidenció una hemorragia subaracnoidea en la convexidad de ambos hemisferios, sin cambios arteriales o venosos, ni aneurismas. **D.** Angiografía por resonancia cerebral con hemorragia subaracnoidea frontal bilateral y parietal izquierda y hallazgos de leucoencefalopatía posterior reversible. **E.** Tomografía computarizada de control en que se evidenció una nueva hemorragia intraparenquimatosa parieto-occipital izquierda. **F-G.** Panangiografía cerebral en la que se observó áreas de adelgazamiento con dilatación de los vasos sanguíneos, principalmente de la circulación posterior y anterior. **H.** Tomografía computarizada de control con nueva área de sangrado occipital izquierdo más superior, con drenaje a ventrículos, importante edema de las cisuras y compromiso principalmente de la fosa posterior

Se le practicó una tomografía computarizada (TC) cerebral simple por sospecha de hemorragia subaracnoidea, cuyos resultados fueron normales (figura 1A). Al regreso del Servicio de Radiología, presentó una nueva crisis que duró dos minutos y se trató con 5 mg intravenosos de diazepam y un bolo de ácido valproico de 20 mg/kg. Se tomó una glucometría en la que se registró un valor de 155 mg/dl.

El examen general fue normal; en cuanto al estado neurológico, la paciente estaba despierta, orientada en las tres esferas, con pupilas reactivas de 3 mm la derecha y de 4 mm la izquierda, con limitación para mirar hacia arriba, sin alteraciones en el fondo de ojo y sin signos focales ni meníngeos.

En el hemograma de ingreso, se reportó un valor de 29.000 leucocitos por mm^3^, 70% de neutrófilos, 25% de linfocitos, 390.000 plaquetas, un índice internacional normalizado (IIN) de 1,03 con tiempo parcial de tromboplastina (TPT) de 28,1-28,6 s, sodio de 140 y potasio de 4 mEq/L (cuadro 1).

**Cuadro 1:** Resultado de los exámenes de laboratorio

Hemograma	Leucocitos: 29.000/mm3 Neutrófilos: 70% Linfocitos: 25% Plaquetas: 390.000
Tiempos de coagulación (s)	TP:10,7/11; INR: 1,03 TPT: 28,1/28,6
Electrolitos (mEq/L)	Sodio: 140 Potasio: 4 Magnesio: 1,69 Calcio: 8,9
Complemento (mg/dl)	C3: 137 C4: 7,6
Anticuerpos	anti-ADN de 13 anti-RNP de 1,5 anti-SSA de 8,9 anti-SSB de 2,5 ANA positivos en 1/160 c-ANCA, p-ANCA: (-)

TP: tiempo de protrombina; INR: *International Normalized Ratio*; TPT: tiempo parcial de tromboplastina; anti-RNP: anticuerpos antirribonucleoproteína; anti-SSA: anticuerpos antisíndrome de Sjögren relacionados con el antígeno A; anti-SSB: anticuerpos antisíndrome de Sjögren relacionados con el antígeno B; ANA: anticuerpos antinucleares: ANCA: *Antineutrophil cytoplasmic antibodies*

Ante la sospecha de trombosis de senos venosos cerebrales o disección vascular arterial, y dada la cefalea “en trueno”, las crisis convulsivas y los sutiles hallazgos neurológicos focales, se tomó una resonancia magnética (RM) cerebral a las 18 horas del ingreso, la cual reveló una hemorragia subaracnoidea en la convexidad de ambos hemisferios, sin cambios arteriales o venosos ni aneurismas (figuras 1, B y C).

La paciente fue trasladada a la unidad de cuidados intensivos, donde ingresó con una presión arterial de 118/83 mm Hg. Se inició la administración de 300 mg de oxcarbazepina cada 12 horas y clonazepam en caso de crisis. Al segundo día de la admisión, registraba 11.100 leucocitos por mm^3^ en el hemograma y no había deterioro de su función neurológica, pero su cefalea persistía, por lo que se inició la administración de hidromorfona.

Se tomó una angiografía por resonancia cerebral, en la cual se evidenció hemorragia subaracnoidea frontal bilateral y parietal izquierda, con hallazgos de leucoencefalopatía posterior reversible (figura 1D), por lo que se reinició el tratamiento con 40 mg de propranolol cada 12 horas. Al cuarto día, persistía la fotofobia sin otros síntomas asociados. Entonces, se solicitaron pruebas de antifosfolípidos, IgG, IgM, perfil de antígeno nuclear extraíble total, anticuerpos anticitoplasma de neutrófilos c-ANCA y p-ANCA, anticuerpos anti-ADN y anticuerpos antinucleares (ANA), y niveles de complemento.

A las 20 horas, se presentó elevación de la presión arterial, por lo que se inició la administración de labetalol en bolos y se logró llevar la tensión arterial a 150/72 mm Hg. En la madrugada del quinto día, la cefalea aumentó con síntomas visuales de hemianopsia homónima derecha, motivo por el que se le administró analgesia y se ordenó una TC de control, la cual evidenció una nueva hemorragia intraparenquimatosa parieto-occipital izquierda (figura 1E).

Se le retiró el labetalol intravenosa de 1 mg/minuto y se ordenó una panangiografía cerebral, tomando todas las precauciones para la aplicación del medio de contraste yodado debido a su alergia. Se reportó un nuevo pico de dolor asociado con presión arterial de 173/70 mm Hg, por lo que se le aplicó hidromorfona en bolos, pero presentó episodios eméticos repetidos.

Se recibieron los siguientes resultados de laboratorio: complemento C3 de 137 mg/dl; C4 de 7,6 mg/dl; anti-ADN de 13 unidades; anticuerpos anti-RNP de 1,5 U/ml; anti-SSA de 8,9 unidades; anti-SSB de 2,5 unidades; niveles de magnesio de 1,69 mEq/L y de calcio de 8,9 mEq/L.

La panangiografía cerebral reveló áreas de adelgazamiento con dilatación de los vasos sanguíneos, principalmente de la circulación posterior y anterior (figuras 1, F-G). Se decidió iniciar la administración de 60 mg de nimodipino cada cuatro horas, lo que mejoró considerablemente la cefalea. Al sexto día, la cefalea y las náuseas disminuyeron; se reportaron ANA positivos de 1/160 y el magnesio se situó en 2,1 mEq/L; se suspendió la infusión de hidromorfona dada la mejoría clínica.

A pesar de su recuperación, súbitamente tuvo un episodio de somnolencia y anisocoria con pupila derecha de 3 mm e izquierda de 5 mm. Se le tomó inmediatamente una neuroimagen, en la que se apreció una nueva área de sangrado occipital izquierdo más superior, con drenaje a ventrículos, importante edema de las cisuras y compromiso de la fosa posterior principalmente (figura 1H).

La paciente fue intubada, sedada con midazolam y fentanilo, y se le administró un bolo de 2 g/kg de manitol y 24 mg intravenosos de dexametasona. Una hora después, presentó posturas de descerebración del lado izquierdo, hiperventilación y pupilas fijas, sin mejoría con las maniobras de reanimación, incluida la infusión de solución salina hipertónica. En la evaluación de los neurocirujanos, se consideró que la paciente no se beneficiaría de una craniectomía descompresiva.

Al séptimo día se le practicaron dos pruebas de apnea, así como un electroencefalograma, que no registró actividad eléctrica cerebral, por lo que se diagnosticó muerte cerebral y se activó el programa de donación de órganos. La autopsia guiada del sistema nervioso central se hizo después de obtener el consentimiento informado para ambos procedimientos, así como para la publicación del caso.

**Figura 2:**
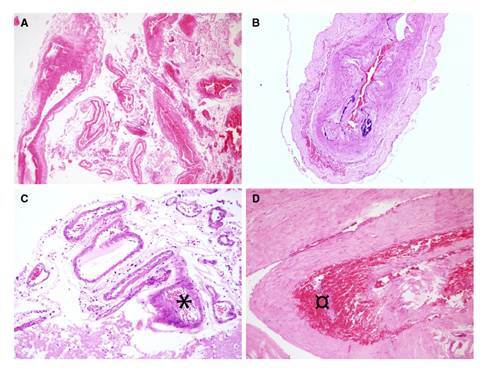
Resultados de la autopsia, microscopía simple. **A.** Cortes de la arteria cerebral posterior que no muestran vasculitis. Hematoxilina y eosina, 10X. **B.** Arteria vertebral normal. Hematoxilina y eosina, 20X. **C.** Vasos arteriales leptomeníngeos sin vasculitis, pero con foco vecino de hemorragia cortical (*). Hematoxilina y eosina, 10X. **D.** Corte de la duramadre con fondo hemorrágico (¤). Hematoxilina y eosina, 20X.

El resultado de la autopsia registró un peso encefálico de 1.600 g (valor normal: 1.250 a 1.400 g), con trombos en el seno longitudinal superior, sin aneurismas en el polígono de Willis, con hemorragias biparietales en la vecindad del seno longitudinal superior, extensa hemorragia témporo-occipital izquierda de 3 x 1,7 x 1,2 cm y otra más lateral de 3 x 2 x 2 cm con extensión al sistema ventricular. No presentaba hemorragias en el tallo cerebral, pero sí en la hipófisis con áreas de infarto. En las arterias se encontró hialinización de la íntima, sin infiltrado inflamatorio ni necrosis fibrinoide, como tampoco depósito de material rojo Congo (figura 2). En el examen de los vasos sanguíneos cerebrales no se encontró vasculitis ni cualquier otra vasculopatía del sistema nervioso central.

## Discusión

El síndrome de vasoconstricción cerebral reversible se caracteriza por una cefalea intensa que puede acompañarse de síntomas neurológicos; en los estudios de neuroimágenes se evidencia contracción segmentaria de las arterias cerebrales que se resuelve de forma espontánea en tres meses, aproximadamente (13).

Suele ser benigno y tiene una baja tasa de reincidencia, por lo que el mal pronóstico y la muerte se han documentado en pocas ocasiones, siendo el grado de vasoconstricción un valor predictivo de complicaciones (14). Entre el 78 y el 90% de los individuos tiene un excelente pronóstico (11,15,16), aunque, aproximadamente, un tercio de los pacientes puede desarrollar accidentes isquémicos o hemorrágicos o edema cerebral reversible. El 71% de los pacientes no evidencia ningún grado de discapacidad y el 29% queda con discapacidades (10). A pesar de su curso benigno, en los estudios de series de pacientes se ha documentado una mortalidad entre el 2 y el 5% (11,15). 

Aunque la fisiopatología no es clara, se han postulado diferentes teorías, entre ellas, la alteración del tono arterial por disfunción del sistema parasimpático, la disfunción endotelial y de las células T que median la vasoconstricción e, incluso, factores inmunológicos y bioquímicos que generan un aumento abrupto de la tensión arterial y estrés oxidativo, así como la influencia de hormonas como el estrógeno (14,17).

El síndrome se presenta más comúnmente en mujeres entre los 20 y los 50 años de edad (18,19), en personas con antecedentes de migraña, así como en quienes usan inhibidores selectivos de la recaptación de serotonina (20). La presencia de vasoconstricción se ha descrito durante el embarazo tardío y entre la primera y tercera semanas posteriores al parto (4,16,21), por lo que el embarazo parece ser la condición clínica más comúnmente relacionada y, la primera semana después del parto, la etapa de mayor riesgo, reportándose en esta una mayor tasa de mortalidad (2).

En este caso, se trató de una mujer hispana de 49 años con migraña como único antecedente, quien tuvo una evolución tórpida que rápidamente progresó hasta el desenlace fatal. La presentación clínica más frecuente es la cefalea intensa reciente o cefalea “en trueno” (10), siendo este el primer síntoma anotado en la mayoría de pacientes (22). La paciente presentó cefalea “en trueno” de difícil control relacionada con picos de hipertensión arterial sistémica.

Los síntomas focales asociados con la localización de la vasoconstricción o la lesión vascular (accidente cerebrovascular, edema focal y hemorragia) ocurren entre el 8 y el 43% de los pacientes (11), en tanto que, en los sujetos con desenlace fatal, los síntomas focales suelen ser más comunes en la enfermedad monofásica y representan un marcador de mal pronóstico (12,16). La progresión rápida del síndrome es extremadamente inusual, pero los hallazgos de la TC y, posteriormente, de la angiografía convencional, pueden llegar a confirmar una vasoconstricción cerebral grave en algunos casos, lo que se asocia con un mal pronóstico o con el curso fatal (16).

En general, las imágenes de diagnóstico inicial es la TC por su facilidad de acceso en los servicios de urgencias y por su utilidad para descartar hemorragias cerebrales (3), siendo la mayoría de resultados normales (21-24). Otra modalidad de imagen diagnóstica que se usa con frecuencia es la resonancia magnética simple o con contraste, la cual ayuda a diferenciar procesos inflamatorios o infecciosos, así como una disección arterial cervical (condición asociada con este síndrome) (3). La prueba de referencia en esta condición es la angiografía de sustracción digital, con una sensibilidad del 100% (14), la cual permite visualizar especialmente la vasoconstricción, por lo que debe reservarse para los casos más sospechosos o cuando hay un empeoramiento del cuadro clínico (3), aunque los resultados pueden ser negativos en las fases iniciales (25).

A pesar de las múltiples opciones diagnósticas, algunos pacientes no llegan a presentar alteraciones (3). No obstante, puede haber hallazgos anormales, siendo los más comunes la hemorragia subaracnoidea de la convexidad, la hemorragia cerebral, el infarto o el edema (23). En cuanto a la resonancia magnética cerebral, esta revela hemorragia subaracnoidea en la convexidad en 20 a 25% de los pacientes, con hemorragia intracerebral parenquimatosa en 6 a 10% de ellos (24). Debido a la hipoperfusión y al curso tardío de la enfermedad, los infartos cerebrales se evidencian en territorios limítrofes entre la circulación anterior y la posterior (21). La resonancia inicial en este caso evidenció una hemorragia subaracnoidea de la convexidad cerebral, sin aneurisma; la angiografía por resonancia tampoco reveló lesiones visibles a pesar de los síntomas visuales y las crisis convulsivas. Por otra parte, la angiografía convencional mostró áreas de constricción y dilatación en la circulación anterior y la posterior (figuras 1, F  y G).

Los casos que no se pueden confirmar por la presencia de signos de constricción en la angiografía, y dado el rápido declive en los casos fatales, son confirmados mediante la biopsia cerebral (23,26); o los hallazgos en la autopsia que excluyan otras condiciones como vasculitis, trombosis de las venas cerebrales o infecciones (24,27). En esta paciente, se hizo una autopsia del sistema nervioso central pensando que su enfermedad de base fuera una vasculopatía no inflamatoria ni sistémica, como es el caso de la vasoconstricción cerebral reversible.

El síndrome de vasoconstricción cerebral reversible puede confundirse con otras arteriopatías, principalmente con la angeítis primaria del sistema nervioso central, por su similitud en manifestaciones clínicas de cefalea, accidente cerebrovascular y alteraciones en la angiografía. Es importante establecer el diagnóstico desde las fases tempranas, ya que el tratamiento puede modificar el curso de estas enfermedades.

En Boston, Rocha EA, *et al*., desarrollaron el puntaje RCVS_2_, que va desde -2 hasta +10, como ayuda para el diagnóstico del síndrome de vasoconstricción cerebral reversible. Para obtenerlo, se tienen en cuenta los siguientes factores: cefalea “en trueno”, única o recurrente; compromiso de la arteria carótida supraclinoidea; factor desencadenante de la vasoconstricción; sexo, y hemorragia subaracnoidea. Se considera que un puntaje de 5 o más tiene una especificidad de 99% y una sensibilidad de 90% para diagnosticar este síndrome (28).

Los pacientes con desenlace fatal usualmente tienen un curso clínico diferente, con síntomas que progresan rápidamente, en lapsos de horas a días, e incluyen cefalea, signos focales y síntomas visuales, imágenes diagnósticas anormales tempranas con signos de infarto, edema cerebral simétrico y constricción arterial difusa, grave, segmentaria y multifocal. Algunos factores asociados con mal pronóstico, son el uso de glucocorticoides, el tratamiento intraarterial con vasodilatadores o la detección de infarto en la primera neuroimagen, así como signos de focalización y una progresión clínica rápida (29).

En la actualidad, no hay un tratamiento aceptado para este síndrome debido a la falta de estudios aleatorizados y a que, generalmente, los pacientes tienen una recuperación completa que no requiere ninguna intervención terapéutica. Sin embargo, se recomienda descontinuar los medicamentos vasoactivos, usar bloqueadores de los canales de calcio, magnesio, no utilizar esteroides, así como evitar la hipotensión. En los grandes estudios retrospectivos, se ha documentado, específicamente, que el tratamiento con esteroides se asocia con empeoramiento clínico, radiológico y angiográfico (29).

## Conclusión

Los casos graves de síndrome de vasoconstricción cerebral reversible suelen ser poco frecuentes, aunque se han reportado algunos en los que se presentan secuelas importantes e, incluso, la muerte. Estos casos suelen tener la misma presentación clínica que los de curso benigno, es decir, cefalea “en trueno” en mujeres en embarazo o en puerperio y hemorragia subaracnoidea de la convexidad cerebral. Sin embargo, algunas características pueden predecir resultados menos favorables, como focalización, alteraciones en la neuroimagen inicial y declive clínico rápido. Aunque tales características no son específicas, es necesario plantear desde el primer momento la necesidad de un seguimiento más estricto para, además, brindar un tratamiento preventivo que disminuya el riesgo de tener mayores complicaciones.

## References

[1] Choi HA, Lee MJ, Chung C-S (2017). Cerebral endothelial dysfunction in reversible cerebral vasoconstriction syndrome: A case-control study. J Headache Pain.

[2] Pilato F, Distefano M, Calandrelli R (2020). Posterior reversible encephalopathy syndrome and reversible cerebral vasoconstriction syndrome: Clinical and radiological considerations. Front Neurol.

[3] Burton TM, Bushnell CD (2019). Reversible cerebral vasoconstriction syndrome: A diagnostic imaging review. Stroke.

[4] Cappelen-Smith C, Calic Z, Cordato D (2017). Reversible cerebral vasoconstriction syndrome: Recognition and treatment. Curr Treat Options Neurol.

[5] Koopman K, Teune LK, Laan M, Uyttenboogaart M, Vroomen PC, De Keyser JD (2008). An often unrecognized cause of thunderclap headache: Reversible cerebral vasoconstriction syndrome. J Headache Pain.

[6] Miller TR, Shivashankar R, Mossa-Basha M, Gandhi D (2015). Reversible cerebral vasoconstriction syndrome, part 1: Epidemiology, pathogenesis, and clinical course. Am J Neuroradiol.

[7] Bouvy C, Ackermans N, Maldonado-Slootjes S, Rutgers MP, Gille M (2020). Reversible cerebral vasoconstriction syndrome revealed by fronto-callosal infarctions. Acta Neurol Belg.

[8] Lee SH, Yun SJ, Choi YH (2017). Reversible cerebral vasoconstriction syndrome presenting as subarachnoid hemorrhage: A rare cause of postpartum seizure. Am J Emerg Med.

[9] Chen SP, Yang AC, Fuh JL, Wang SJ (2013). Autonomic dysfunction in reversible cerebral vasoconstriction syndromes. J Headache Pain.

[10] Calabrese LH, Dodick DW, Schwedt TJ, Singhal AB (2007). Narrative review: Reversible cerebral vasoconstriction syndromes. Ann Intern Med.

[11] Singhal AB, Hajj-Ali RA, Topcuoglu MA, Fok J, Bena J, Yang D (2011). Reversible cerebral vasoconstriction syndromes: Analysis of 139 cases. Arch Neurol.

[12] Robert T, Kawkabani Marchini A, Oumarou G, Uske A (2013). Reversible cerebral vasoconstriction syndrome identification of prognostic factors. Clin Neurol Neurosurg.

[13] Lozupone E, Distefano M, Calandrelli R, Marca GD, Pedicelli A, Pilato F (2020). Reversible cerebral vasoconstriction syndrome: A severe neurological complication in postpartum period. Neurol India.

[14] Abadía L, Castañeda C, Méndez JA, Coral J, Zarco LA (2014). Síndrome de vasoconstricción cerebral reversible: revisión de tema. Universitas Médica.

[15] Topcuoglu MA, Singhal AB (2016). Hemorrhagic reversible cerebral vasoconstriction syndrome: Features and mechanisms. Stroke.

[16] Suchdev K, Norris G, Zak I, Mohamed W, Ibrahim M (2018). Fulminant reversible cerebral vasoconstriction syndrome. Neurohospitalist.

[17] Enríquez P, Ariza-Varón M, Enríquez MN, Navarro CE (2020). Síndrome de vasoconstricción cerebral reversible inducido por maniobra de Valsalva: reporte de caso y revisión de la literatura. Acta Neurológica Colombiana.

[18] Pop A, Carbonnel M, Wang A, Josserand J, Auliac SC, Ayoubi J-M (2019). Posterior reversible encephalopathy syndrome associated with reversible cerebral vasoconstriction syndrome in a patient presenting with postpartum eclampsia: A case report. J Gynecol Obstet Hum Reprod.

[19] Singhal AB (2004). Cerebral vasoconstriction syndromes. Top Stroke Rehabil.

[20] Kunchok A, Castley HC, Aldous L, Hawke SH, Torzillo E, Parker GD (2018). Fatal reversible cerebral vasoconstriction syndrome. J Neurol Sci.

[21] Singhal AB, Bernstein RA (2005). Postpartum angiopathy and other cerebral vasoconstriction syndromes. Neurocrit Care.

[22] Ducros A (2012). Reversible cerebral vasoconstriction syndrome. Lancet Neurol.

[23] Sattar A, Manousakis G, Jensen MB (2010). Systematic review of reversible cerebral vasoconstriction syndrome. Expert Rev Cardiovasc Ther.

[24] Williams TL, Lukovits TG, Harris BT, Harker Rhodes C (2007). A fatal case of postpartum cerebral angiopathy with literature review. Arch Gynecol Obstet.

[25] Ducros A, Bousser M-G (2009). Reversible cerebral vasoconstriction syndrome. Pract Neurol.

[26] Ducros A, Wolff V (2016). The typical thunderclap headache of reversible cerebral vasoconstriction syndrome and its various triggers. Headache.

[27] Calado S, Vale-Santos J, Lima C, Viana-Baptista M (2004). Postpartum cerebral angiopathy: Vasospasm, vasculitis or both?. Cerebrovasc Dis.

[28] Rocha EA, Topcuoglu MA, Silva GS, Singhal AB (2019). RCVS(2) score and diagnostic approach for reversible cerebral vasoconstriction syndrome. Neurology.

[29] Valencia-Mendoza M, Ramírez-Rodríguez N, Vargas-Ávila N, Peña-Ortiz A, Corzo-Villamizar M, Serna-Ramírez L (2019). Fatal reversible cerebral vasoconstriction syndrome: A systematic review of case series and case reports. J Clin Neurosci.

